# Large-scale spontaneous self-organization and maturation of skeletal muscle tissues on ultra-compliant gelatin hydrogel substrates

**DOI:** 10.1038/s41598-020-69936-6

**Published:** 2020-08-06

**Authors:** Joen H. Jensen, Selgin D. Cakal, Jingwen Li, Christian J. Pless, Carmen Radeke, Morten Leth Jepsen, Thomas E. Jensen, Martin Dufva, Johan U. Lind

**Affiliations:** 1 Department of Health Technology, Technical University of Denmark, Building 423, 2800 Kgs. Lyngby, Denmark; 2 Section of Molecular Physiology, Department of Nutrition, Exercise and Sports, University of Copenhagen, 2100 København Ø, Denmark; 3 The Danish National Research Foundation and Villum Foundation’s Center for Intelligent Drug Delivery and Sensing Using Microcontainers and Nanomechanics (IDUN), Technical University of Denmark, Building 423, 2800 Kgs. Lyngby, Denmark

**Keywords:** Biomaterials, Tissues, Musculoskeletal models, Muscle contraction, Biomedical engineering, Gels and hydrogels, Biomaterials, Biomaterials - cells, Tissue engineering

## Abstract

Cellular self-organization is the fundamental driving force behind the complex architectures of native tissue. Yet, attempts at replicating native tissue architectures in vitro often involve complex micro-fabrication methods and materials. While impressive progress has been made within engineered models of striated muscle, the wide adaptation of these models is held back by the need for specific tools and knowhow. In this report, we show that C2C12 myoblasts spontaneously organize into highly aligned myotube tissues on the mm to cm scale, when cultured on sufficiently soft yet fully isotropic gelatin hydrogel substrates. Interestingly, we only observed this phenomenon for hydrogels with Young’s modulus of 6 kPa and below. For slightly more rigid compositions, only local micrometer-scale myotube organization was observed, similar to that seen in conventional polystyrene dishes. The hydrogel-supported myotubes could be cultured for multiple weeks and matured into highly contractile phenotypes with notable upregulation of myosin heavy chain, as compared to myotubes developed in conventional petri dishes. The procedure for casting the ultra-soft gelatin hydrogels is straight forward and compatible with standardized laboratory tools. It may thus serve as a simple, yet versatile, approach to generating skeletal muscle tissue of improved physiological relevance for applied and basic research.

## Introduction

Skeletal muscle comprises the largest tissue in the human body, and plays an essential role in locomotion as well as in metabolism and homeostasis^1,2^. The diseases associated with skeletal muscle are wide ranging and includes myopathies^3^, dystrophies as well as insulin resistance in diabetes mellitus type 2^4^. Within in vitro research, traditional tissue culture polystyrene (TCPS) remains the most widespread substrate for culturing and studying skeletal muscle in health and disease. The use of TCPS is common, despite the general recognition that the practically incompressible and planar TCPS dishes constitute a very poor representation of the native cellular environment. Aiming to overcome this paradox, numerous advanced in vitro models of striated muscle have emerged in recent years. These models include freestanding micro tissues^5–10^, bio-printed constructs^11,12^, 3D cultures in fibrous scaffolds^13,14^ and microphysiological systems^3,15,16^. These novel approaches generally display highly unidirectional muscle tissues, and native function in terms of concerted contractile shortening. Nevertheless, there are several obstacles to the widespread adaptation of these models. For instance, many custom solutions are not readily compatible with standard laboratory tools and analyses, and their fabrication may require highly specific tools and detailed expertise.

In the vast majority of engineered models of striated muscle, extracellular bio-chemical or mechanical cues are applied for guiding cellular organization into aligned tissues, for instance in the form of nanofibrous scaffolds^13,14,17^. Alternatively, in freestanding micro-tissues, external attachment points gives rise to an anisotropic force along an extended tissue^5,6^. On planar yet deformable substrates, micro contact-printed line-patterns of extracellular matrix (ECM) proteins as well as micro-molded ridges have been applied to generate highly aligned laminar muscle tissues^3,18–20^. For instance, Engler et al*.* applied microcontact printing of Collagen I lines on polyacrylamide gels to generate aligned C2C12 myotubes and determine the optimal substrate stiffness for maturing these myotubes^20^. Here they found that the most mature myotubes occur at gels with physiological Young’s modulus of ~ 12 kPa as indicated by the degree of striations^20^. Similarly, Bettadapur and co-workers molded ~ 2 µm × 5 µm × 35 µm ridges on the surface of enzymatically cross-linked gelatin hydrogels with Young’s modulus in the 30–50 kPa range, to generate unidirectional, extended C2C12 myotubes^19^. Across the diverse approaches, the formation of aligned tissues is closely tied to self-organization, where individual cells respond to the cues arising from neighboring cells. Indeed, even on traditional isotropic TCPS substrates, C2C12 myotubes align locally in domains of up to ~ 0.4 mm^2^, as documented by Junkin et al*.*^21^.

In this study, we show that the ability of C2C12 myoblasts to self-organize increases considerably when applying ultra-soft gelatin hydrogel substrates, with Young’s modulus in and below the range of the native microenvironment^20,22^. On fully isotropic substrates, skeletal myoblasts were found to spontaneously arrange and fuse into highly aligned myotube domains spanning ~ 100 mm^2^. After ~ 2 weeks of culture, these myotubes become highly contractile with pronounced upregulation of both slow myosin heavy chain type I (MyHC-I) and fast myosin heavy chain type II (MyHC-II). The method presented here may thus serve as a simple yet versatile basis for engineering physiologically relevant skeletal muscle tissues within standard laboratory tools.

## Results

### Comparing spontaneous C2C12 organization on isotropic gelatin hydrogels and TCPS

**Figure 1:**
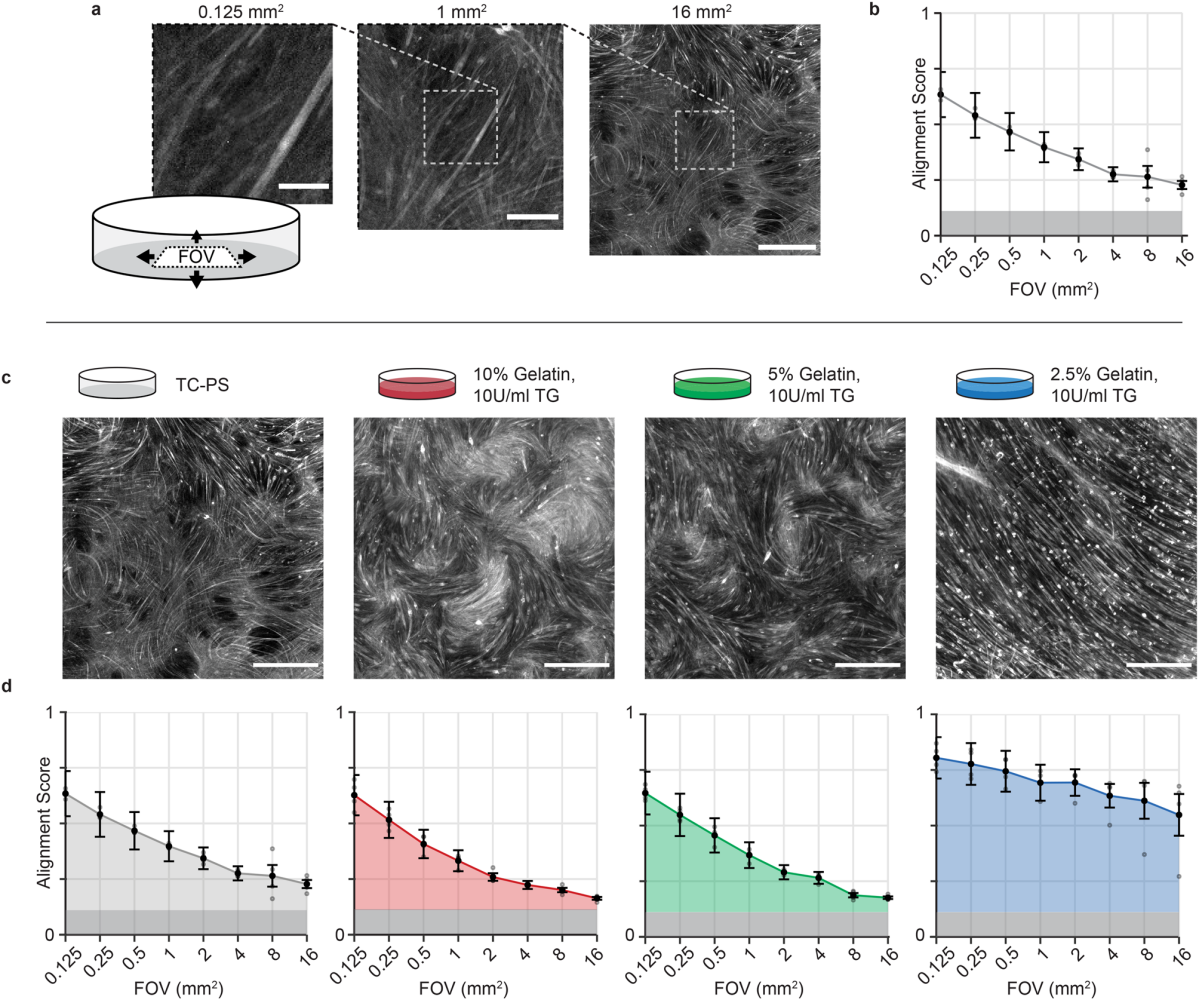
Local and global spontaneous myotube alignment. C2C12 myoblasts were cultured on TC polystyrene and stained for F-actin after 9 days of differentiation. **(a)** Sample images (of n = 4) show that in conventional cell culture the degree of cellular alignment decreases with increasing field of view (FOV). Scale-bars: (left to right) 100 µm, 250 µm, 1 mm **(b)** Myotube alignment was quantified from F-actin images using the ImageJ Plug-in, OrientationJ, and given as an alignment score (0–1) for multiple FOVs ranging from 0.125 mm^2^ to 16 mm^2^. Areas shaded in dark grey indicate values below theoretical point of random alignment (0.11) for the analysis method **(c)** Large FOV images (16 mm^2^, scale bars = 1 mm) of C2C12 myotubes on various substrates at day 9 of differentiation. Left to right: TC polystyrene, 10% w/v gelatin—10 U/mL Transglutaminase (TG), 5% w/v gelatin—10 U/mL TG and 2.5% w/v gelatin—10 U/mL TG. **(d)** Alignment score as a function of FOV for C2C12 myotubes developed on various substrates. Left to right: TC polystyrene, 10% w/v gelatin—10 U/mL TG, 5% w/v gelatin—10 U/mL TG and 2.5% w/v gelatin—10 U/mL TG. Points indicate mean ± s.e.m. (n = 4). Evaluation performed on day 9 after initiation of differentiation.

Tissue-level self-organization is driven by intercellular feedback as well as cues from the external environment. Given that C2C12 myoblasts locally self-organize even on conventional isotropic TCPS^21^, we hypothesized that on highly deformable substrates, more extensive self-organization, may occur. To investigate this hypothesis, we compared C2C12 culture on TCPS to cultures on gelatin hydrogels cross-linked with transglutaminase (TG), which can be formulated with Young’s Modulus as low as single kPa^23^. In order to quantify the degree of cellular alignment across samples, we utilized the ImageJ Plug-In, OrientationJ^24^ to evaluate the distribution of cellular alignments and generate an alignment score (0–1) equivalent to 0–100% of the signal falling within the same 20°. In all samples, fluorescent images of cellular F-actin were applied as input. To compare local vs global scale organization, we analyzed incrementally larger field of views (FOV) spanning ~ 350 µm × 350 µm (0.125 mm^2^) to 4 mm × 4 mm (16 mm^2^), see Fig. 1a. Using this approach, we observed a rapid decline in the degree of alignment for C2C12 myotubes on TCPS as FOV increases, in agreement with previous work^21^, see Fig. 1b. Subsequently, we investigated spontaneous myotube alignment on 2.5, 5 and 10% w/v gelatin hydrogels cross-linked with 10 U/mL TG. Interestingly, despite being several orders of magnitude softer than TCPS, hydrogels based on 10 and 5% w/v gelatin, gave rise to cellular self-organization properties that were close to identical to those observed for TCPS, see Fig. 1c,d. For the largest FOVs, the degree of alignment was even lower than that observed on TCPS. However, when gelatin content was lowered further to 2.5% w/v we observed a striking change. Even in the largest FOV of 16mm^2^, more than 50% of the culture was oriented within 20°. This is comparable to the degree of alignment observed only for the smallest, most local FOVs for the other conditions. Further, the local organization was also increased for the 2.5% w/v gelatin, as more than 75% of the culture fell within 20° for the smallest FOV in the analysis.

### The mechanical properties of soft gelatin hydrogels regulate C2C12 self-organization

**Figure 2:**
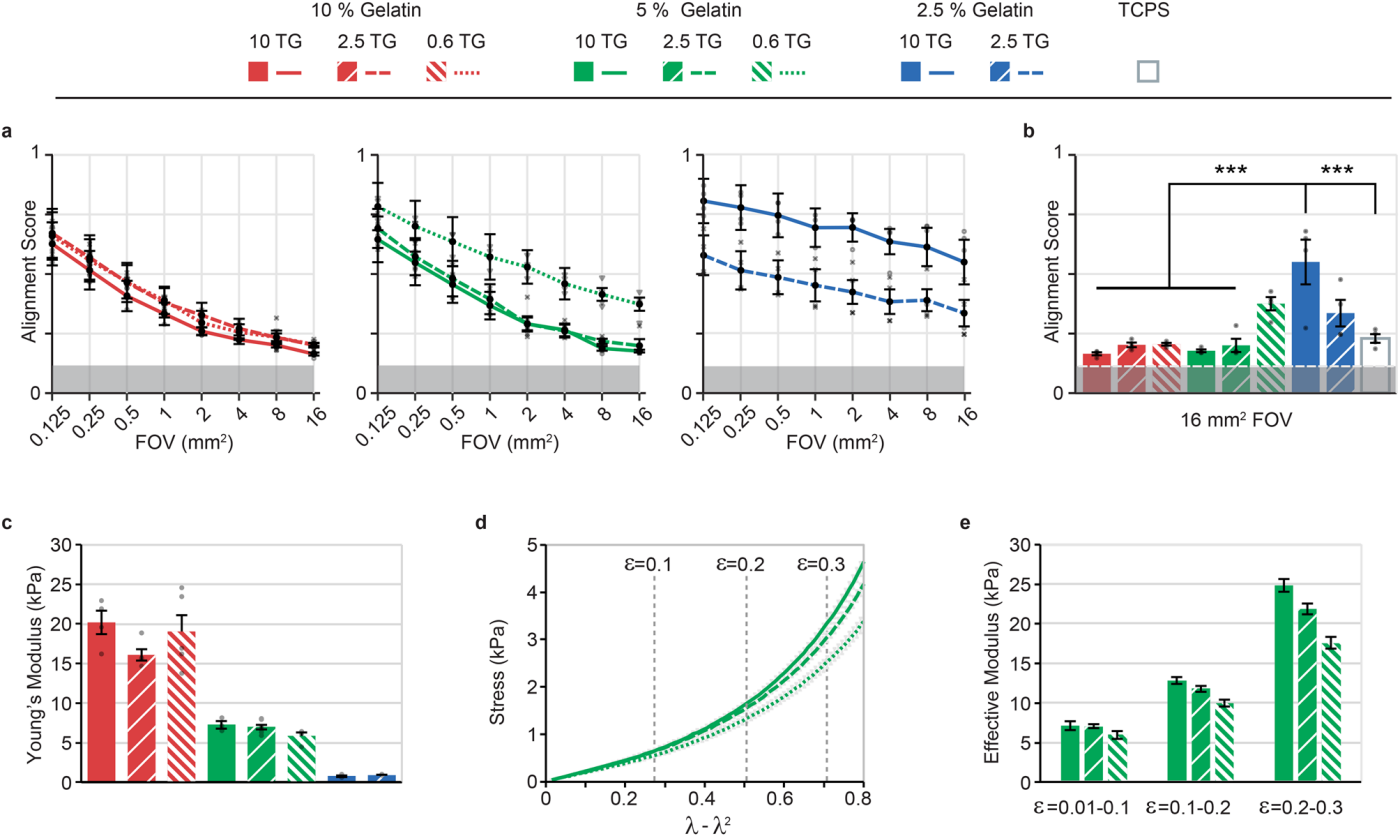
Substrate mechanical properties determine spontaneous myotube alignment. (**a**) Alignment score as a function of FOV for C2C12 cultures developed on a range of gelatin hydrogels varying in both gelatin concentration (color): 2.5–10% w/v , and transglutaminase concentration (TG) (pattern): 0.6–10 U/ml. Error bars indicate mean ± s.e.m. (n = 4). Evaluation performed on day 9 after initiation of differentiation. **(b)** Comparison of the alignment score for the largest field of view (16 mm^2^) for each of the analyzed conditions. Error bars indicate mean ± s.e.m. (n = 4). Statistical significance: ***P < 0.001. **(c)** Young’s modulus for each hydrogel compositions analyzed in (a-b), error bars indicate mean ± s.e.m., for all conditions n ≥ 3 **(d)** Stress vs. strain equivalent for 5% w/v gelatin hydrogels cross-linked with 0.6, 2.5 or 10 U/ml TG, under compression. Less strain stiffening is observed for lower degrees of enzymatic cross-linking. Vertical lines indicate 0.1, 0.2 and 0.3 engineering strain (ε) **(e)** Approximate effective modulus for 5% w/v gelatin hydrogels (0.6–10 U/ml TG) in strain regimes 0.01–0.1, 0.1–0.2 and 0.2–0.3 engineering strain (ε).

The mechanical properties of enzymatically cross-linked gelatin hydrogels is influenced by both the solid protein content, gelatin bloom, and extent of covalent cross-linking induced by TG^23^. To investigate the influence of enzymatically introduced covalent cross-linking, we varied the TG concentrations between 10, 2.5 and 0.6 U/ mL, for hydrogels with 10, 5 and 2.5% w/v gelatin content, see Fig. 2. 2.5% w/v gelatin cross-linked with 0.6 U/mL TG failed to form a stable hydrogel. The remaining eight hydrogel compositions were stable and were used as substrate for C2C12 culture. Following proliferation and differentiation into myotubes, we evaluated local and global alignment, for each of the gel compositions, see Fig. 2a,b. We observed a clear global-scale myotube self-organization only for selected compositions: 5% w/v gelatin—0.6 U/ml TG, 2.5% w/v gelatin—10 U/ml TG and 2.5% w/v gelatin—2.5 U/ml TG. For all other compositions, the self-organization was highly similar to that observed for conventional TCPS. These distinct types of organization—only local vs. global—appeared at the onset of myotube formation, see supplementary Fig. 1.

For each of the hydrogel compositions, we next estimated their Young’s modulus at ambient conditions, summarized in Fig. 2c. As expected, the stiffness generally increased with increasing gelatin and TG concentrations, with the gelatin solid content having the largest impact on the nominal Young’s modulus. Notably, the three samples that gave rise to global self-organization were the three softest compositions, which all displayed Young’s modulus ≤ 6 kPa. While the three compositions based on 5% w/v gelatin displayed similar Young’s modulus of ~ 7.1, ~ 7 and ~ 6 kPa, we only observed large-scale alignment for the formulation with the lowest amount of TG. Interestingly, this formulation was notably less strain-stiffening, see Fig. 2d,e, which can have significant influence on cellular responses^25,26^. Based on our observations, we performed a secondary screening of hydrogels in the range of 3–5% w/v gelatin with 0.6 or 0.3 U/mL TG, see supplementary Fig. 2. This confirmed that large-scale self-organization occurs in this range, given the appropriate amount of covalent cross-linking. We thus conclude that several gel formulations can be made to yield isotropic substrates where large-scale self-organization occur, by balancing gelatin concentrations below 5% w/v with an appropriate cross-linking degree. A Young’s modulus of 6 kPa appears to be the upper threshold. To ensure that our observations were not tied to the particular strain of the C2C12 cell line, we performed a control experiment culturing a separate C2C12 line on 2.5% w/v gelatin with 10U/mL TG. Indeed, this also displayed large-scale spontaneous alignment, see supplementary Fig. 3. Lastly, it is worth noting that for very low TG concentrations of 0.3 U/ml, gel stability was diminished, and several layers of cells were observed. A similar behavior was observed for 0.6 U/ml TG, in particular for gelatin concentrations ≤ 3% w/v.

**Figure 3:**
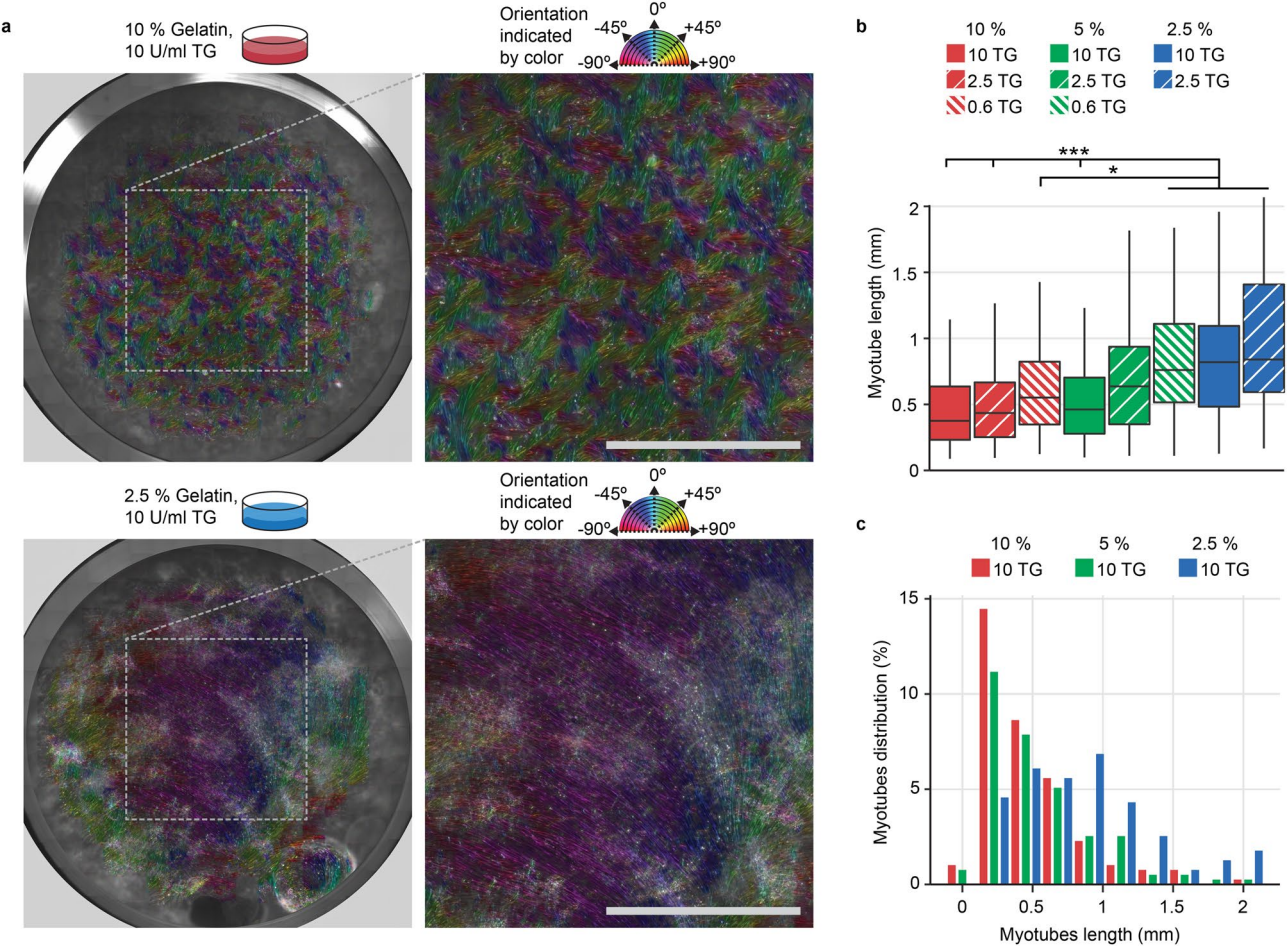
Spontaneous global organization increases myotube lengths. (**a**) Representative composite image (n = 4) of F-actin in C2C12 myotubes, spanning a full well in a standard 12-well plate. Composite images were composed of 252 frames, and the OrientationJ ImageJ plug-in was used to false-color the image according to the local angle of orientation of F-actin filaments. *Top:* C2C12 culture on 10% w/v gelatin—10 U/mL TG hydrogels. *Bottom:* C2C12 culture on 2.5% w/v gelatin—10 U/mL TG hydrogels. Close-up areas: 1cm^2^, scale bars 5 mm **(b)** Length of myotubes developed on a range of hydrogel substrates: gelatin: 2.5–10% w/v, TG: 0.6–10 U/ml. Middle bar represents median length, while the upper and lower bound of the box respectively represents the upper and lower quartile. Lines indicate largest and lowest value within 1.5 times the interquartile range. For each gel composition, four separate cultures were used to evaluate at least 100 random myotubes. Statistical significance: *P < 0.05, ***P < 0.001 **(c)** Distributions of myotube lengths for 2.5, 5 and 10% (w/v) gelatin hydrogels, cross-linked using 10 U/mL TG. Evaluation performed at day 9 after initiation of differentiation.

### Large-scale self-organization increases C2C12 myotube length

Native adult myotubes span from several mm to cm in length. In contrast, engineered C2C12 myotubes developed in conventional culture plates rarely exceed 0.5 mm due to their lack of organization^21^. Therefore, for the soft gelatin substrates that gave rise to spontaneous self-organization, we hypothesized that longer myotubes would simultaneously be obtained. To evaluate the degree of the self-organization, we imaged several full wells in a 12-well plate comparing a high 10% w/v and low 2.5% w/v gelatin hydrogel each cross-linked using 10 U/ml TG,see Fig. 3a,b, and supplementary Fig. 4. We applied the ImageJ OrientationJ plugin to identify and false-color images according to the local angular orientation of the F-actin filaments. As anticipated, we observed large-scale organization for all 2.5% w/v gelatin formulations, while for all 10% w/v gelatin we found distinct ~ 500 µm-sized locally organized domains. Interestingly, for the soft formulation, the large-scale alignment appeared to be influenced significantly by the edges of the well, while one or a few orientations would dominate towards the center. This indicates that the degree of organization could be limited by the presence of external walls. Next, we evaluated the myotubes length on each of the previously investigated gel formulations at day 9 of differentiation. Here, we observed a drastic increase in median myotube length from 0.3 mm to 0.8 mm from the stiffest to softest gel formulation, with myotube lengths frequently reaching 2 mm see Fig. 3b,c. Notably, the measured lengths were restricted by the boundaries of the 3.4 mm × 2.7 mm FOV used in the analysis. Indeed, when extending differentiation time to 21 days and FOV to 15.8 mm × 11.9 mm, we observed several myotubes spanning > 5 mm, thus approaching their natural lengths^27^, see supplementary Fig. 5.

**Figure 4:**
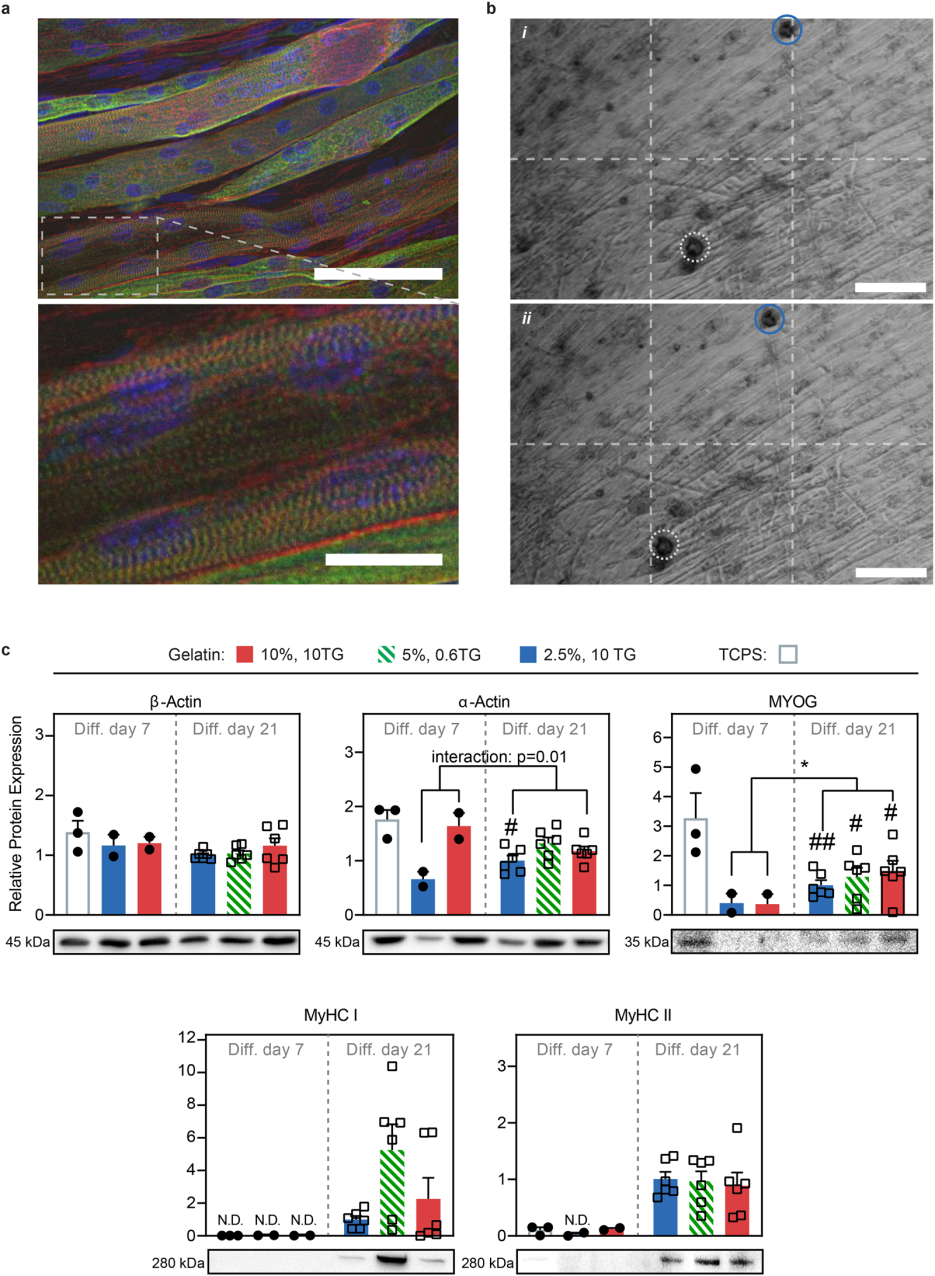
Long term C2C12 culture on compliant gelatin hydrogels generates myotubes of high contractile maturity. (**a**) Confocal images of immunostained myotubes at day 21 on 2.5% w/v gelatin—10 U/ml TG substrate. Blue: DAPI, Red: Actin, Green: α-actinin. Scale bars, top: 100 µm, Bottom: 25 µm (b) Bright field microscopy images of spontaneously contracting myotubes generated on 2.5% gelatin—10 U/ml TG hydrogels, at day 12 of differentiation. Grid interval: 0.5 mm, scale-bar 250 µm. Inserted circles mark immediately traceable points, and their displacement upon contraction. Corresponding videos can be found in the supplementary information (c) Western-blot evaluation of selected myotube proteins across various substrates and time-points. For each protein, the expression relative to that obtained for day 21 of differentiation on the 2.5% gel is shown. Error bars indicate mean ± s.e.m. Corresponding representative western blots are depicted below each graph. Full gel images can be found in the supplementary information. Two statistical analysis were performed: *i)* Two-way ANOVA followed by Sidak’s post hoc test for 2.5% and 10% samples at days 7 and 21, Statistical significance indicator: *P < 0.05 significant difference between day 7 and day 21 differentiation *ii)* One-way ANOVA followed by pairwise Tukey’s post hoc test for multiple comparison between samples (n = 2 samples excluded).Statistical significance indicators: #*P* < 0.05, ##*P* < 0.01, significant difference with day 7 TCPS.

### Long-term culture of C2C12 myotubes on compliant gelatin improves their contractile maturity

Having established that C2C12 myoblasts can self-assemble into aligned and extended myotubes on compliant gelatin substrates, we next sought to evaluate the functional maturity of these myotubes. An important benefit of applying compliant substrates is that these allow extended culture duration of myotubes^19^, while myotubes tend to delaminate after approximately a week of differentiation on conventional TCPS. Thus, we monitored the functional maturity of myotubes developed on the gelatin hydrogel substrates for up to three weeks of differentiation. We compared three gel compositions: ‘soft’ ~ 1 kPa (2.5% w/v gelatin—10 U/ml TG) and ‘medium’ ~ 6 kPa (5% w/v gelatin—0.6 U/ml TG) where large scale self-organization occur, and ‘hard’ ~ 20 kPa (10% w/v gelatin—10 U/ml TG) gels where no large-scale organization is observed. After 21 days of differentiation, we performed immunohistochemistry and confocal imaging. For all three compositions, this revealed highly fused and striated myotubes, see Fig. 4a and supplementary Fig. 6. Interestingly, after approximately 10 days of differentiation, we observed strong spontaneous contractions, see Fig. 4b and supplementary movies 1–4. These contractions would continue for the remaining culturing period. For the observed samples, see supplementary movies, the contractions appear more concerted on the soft and medium substrates. However, we did not perform a stringent analysis and cannot rule out that these qualitative observations were due to chance. We next compared a number of skeletal muscle markers for myotube samples at day 7 and 21 of differentiation on gel substrates, see Fig. 4c. We further compared these to myotubes differentiated for 7 days on conventional TCPS, the longest reliable maturation time on this substrate. For β-actin, serving as an equal protein loading control, we observed no notable changes in relative expression level across any of the tested substrates and time points. For skeletal muscle α-actin isoform, which is associated with myoblast fusion and muscle formation^28^, we also observed similar expression across all samples, apart from the softest gel sample at day 7, which was notably lower. This could indicate that myoblast fusion was slower on the softest substrates. Similarly, myogenin–a key regulator of myoblast differentiation and fusion^28^– had lower relative expression on both the soft and hard gel substrates at day 7, as compared to TCPS. A similar trend was observed when analyzing these markers prior to fusion at the onset of differentiation, see supplementary Fig. 7, however, after 21 days of differentiation, these became comparable to TCPS. These observations indicate that some aspects of myotube fusion are slower on the softest hydrogel substrates, but reach a similar level to that observed on TCPS after extended culture. Lastly, to evaluate the functional maturity of the myotubes, we compared the expression level of the late-stage maturation markers contractile myosins MyHC-I (slow) and MyHC-II (fast)^29^ for all samples. Interestingly, for all day 7 samples we did not detect expression. In contrast, for all three hydrogel-based substrates we detected both MyHC-I and MyHC-II after 21 days of differentiation, with MyHC-I expression being notably higher on the medium gel formulation.

## Discussion

Our results demonstrate that C2C12 myoblasts spontaneously organize and fuse into aligned myotubes spanning multiple millimeters, when cultured on highly compliant isotropic gelatin hydrogel substrates. This large-scale spontaneous self-organization is closely regulated by substrate stiffness, as we only observed this phenomenon for the most compliant formulations with Young’s Modulus ≤ 6 kPa. Notably, even slightly stiffer gels gave rise to only local self-organization similar to that observed in conventional TCPS petri dishes, which have Young’s Modulus in the GPa range. As expected, we observed no changes in the organization of myotubes after fusion, even after several weeks of culture. This suggests that self-organization occurs either shortly before or during myoblast fusion into myotubes, and logically, that the softer substrates induce larger scale self-organization by improving cellular alignment before and/or during fusion. The detailed mechanism is not clear, and a full investigation would require several further experiments paired with an in-depth expression analysis. Still, a key aspect is likely that the softer gel substrates are more prone to deformation and remodeling by the cells, due to their lower stiffness and lower solid content. Thus, individual cells may introduce local substrate anisotropies, which may in turn induce alignment of neighboring cells^20,30^. An additional potential mechanism could be that soft substrates decrease the speed of cellular fusion, allowing fever initially formed myotubes to dominate tissue directionality. Yet, we did not observe any notable differences in speed of myotube formations across substrates. Thus, the spontaneous self-organization appears to be primarily governed by substrates being sufficiently susceptible to cell-induced deformations.

An important benefit of applying soft gel substrates is to support longer-term culture than possible in regular TCPS dishes where delamination occurs after roughly 7 days of differentiation. Interestingly, when comparing the relative amount of α-actin and myogenin at this time point, these were generally lower for the hydrogel substrates than for TCPS, in particular for the softest gel formulation. This indicates that myotube formation and maturation is initially slowed down on the soft substrates. However, as culturing time on the gels is extended to several weeks, these levels become comparable to TCPS. Furthermore, other signs of myotube maturity such as tissue contraction and expression of contractile myosin heavy chain increased for the gel substrates, to ultimately provide phenotypes of higher physiological relevance than those obtained in conventional culture. Interestingly, MyHC-I (slow) appeared the highest for the 5% gelatin—0.6 U/ml TG medium stiffness gel, which with ~ 6 kPa was the stiffest gel formulation where large-scale self-organization occurred. This could indicate a tradeoff between myotube length and alignment being promoted by softer substrate formulations, and contractile maturation being accelerated on stiffer substrates that provide a larger auxotonic contractile load. The 5%—0.6 U/ml gel also closely matches the mechanical properties of native ECM^22^. Thus, our findings support the notion that in order to faithfully replicate in vivo phenotypes, in vitro tissue engineering should strive to reproduce the native microenvironment ^22^.

Although the fundamental purpose of any in vitro tissue model is to mimic the in vivo counterpart, it is often equally important is that the tissue models are robust, reproducible, scalable, low cost, and readily available. Thus, increasing tissue model complexity may not always be beneficial, if simpler approaches are sufficient for a given purpose. Within muscle tissue models, several approaches have been presented to engineer complex and highly organized tissues, as well as means of evaluating e.g. tissue contractility^5–10,14–16^. Compared to these, the main benefit of the procedure presented here, is its extreme simplicity. No specialized equipment or know-how is required and the substrate casting is a simple procedure. It is therefore fully compatible with most standard laboratory tools. Indeed, our work serves to illustrate how cellular self-assembly combined with minimal adjustments to the microenvironment, can go a long way towards providing more relevant in vitro models of skeletal muscle.

## Materials and methods

### Casting soft gelatin hydrogel substrates

The preparation of hydrogels started with the preparation of two sterile solutions, one containing dissolved gelatin (2X final concentration) and the other containing dissolved TG (2X final concentration). The gelatin solution was prepared by first aliquoting PBS to a 50 ml centrifuge tube. In a fume hood, 0.5% (v/v) Chloroform was added and mixed with the PBS. Subsequently, gelatin powder (48723 Sigma-Aldrich) was added to the solution, and the solution was placed in a 37 °C water bath for at least 30 min until the gelatin was fully dissolved. Once dissolved, the lid was loosened to let chloroform evaporate for 20 min. Once evaporated, the solution was reheated in the 37 °C water bath for 10 min. The TG solution was prepared, while the gelatin solution was dissolving, by mixing TG (ACTIVA TI Transglutaminase—1002–1 K, 100 U/g) with PBS. The TG solution was vortexed until the TG was fully dissolved and then filtered through a 45 µm filter. Once the two solutions had been prepared, they were both heated to 37 °C prior to mixing. Once heated, the two solutions were mixed 1:1, and immediately cast into well plates at 130 µL/cm^2^. After casting, the plate was placed in a refrigerator for 15 min. After refrigeration, the plate was placed in an incubator (37 °C, 100% Humidity, 5% CO_2_) for 4 h to cross-link. After cross-linking, the hydrogels were washed in PBS 3 × 10 min.

### C2C12 cell culture and maturation

The used C2C12 myoblasts were kindly provided by professor Amira Klip, SickKids hospital, Toronto, Canada and were originally from Dr. Nobuharu L. Fujii´s lab Tokyo Metropolitan University, Japan. Myoblasts were cultured using standard sterile technique and incubator (37 °C, 100% Humidity, 5% CO_2_). Prior to seeding on gel substrates, myoblasts were maintained in a 75 cm^2^ tissue culture flask in growth medium (DMEM + 10% FBS + 1% P/S). The culture was passaged every other day upon reaching ~ 90% confluency, passage no. was kept < 10 from provided stock in all experiments. The C2C12 cultures were seeded on freshly prepared hydrogels at a density of 15,000 cells/cm^2^. Importantly, the subculture used for seeding should not be highly confluent (≤ 50%), as cell lumps will disrupt the self-organization of the culture. The cultures were allowed to proliferate in growth medium for 2 days, reaching ~ 90% confluency, after which the culture was rinsed with PBS and switched to differentiation medium (DMEM + 2% Horse Serum + 1% P/S). Medium was changed Mondays, Wednesdays and Fridays, replacing 3/4 of the medium. Control experiment in supplementary information was performed using a separate authenticated C2C12 line (Sigma-Aldrich C3H muscle myoblast, 91092101) at passage 9.

### Myotube staining for alignment quantification

The C2C12 cultures were stained by first washing thrice in PBS for 4 min, then fixed using 3% paraformaldehyde for 30 min, again rinsed thrice in PBS for 4 min, permeabilized for 15 min using 0.05% Saponin/PBS and 1% BSA. The permeabilization solution was removed and samples were incubated with staining solution (1 × Alexa flour 594 Phalloidin and 1 µg/mL Hoechst 33342) for 20 min. Finally, the samples were rinsed thrice in PBS for 4 min and the PBS replaced. Fluorescence microscopy was performed using a Zeiss Observer Z1 microscope with a mounted Zeiss AxioCam Mrm. The imaging software used for capturing fluorescence images was AxioVision (v. 4.8.2.0). Quantification of alignment and false-color image generation was performed using a custom ImageJ macro iterating over the ImageJ Plug-in, OrientationJ. The resulting orientation distribution data were further analyzed using R-studio.

### Western blot preparations and quantification

C2C12 myoblasts or myotubes samples were washed once in ice-cold PBS, and protein extracted using 150 µl per well ice-cold NP-40 lysis buffer containing 0.05 M Tris-Base (pH 7.4), 0.15 M NaCl, 1 mM EDTA, 1 mM EGTA, 0.05 M sodium flouride, 5 mM sodium pyrophosphate, 2 mM sodium orthovanadate, 1 mM benzamidine, 0.5% protease inhibitor cocktail (P8340, Sigma Aldrich), and 1% NP-40. Cell lysates were then centrifuged at 18,320 g for 20 min at 4˚C and protein concentrations were measured using Pierce BCA Protein Assay Kit (23225, Thermo Fisher-Scientific). Diluted samples of equal protein content in sample-buffer (62.5 mM Tris (pH 6.8), 2% SDS, 10% glycerol, 0.1 M DTT, 0.01% bromophenol blue) were heated at 95˚C for 5 min prior to loading. Equivalent amounts of protein were separated by 7–12% SDS-PAGE gels and semi-dry transferred to PVDF membranes (Immobilon-P Transfer Membranes; Millipore). The membranes were blocked in 3% skim milk-TBST (Tris Buffered Saline with Tween) for 30 min at room temperature, followed by overnight incubation with the indicated primary antibodies at 4˚C. The primary antibodies included MYOG (H00004656-D01P, Abnova), MyHC-I (A4-840-C, Developmental Studies Hybridoma Bank (DSHB)), MyHC-II (A4-74-C, DSHB), α-actin (A2066, Sigma), β-actin (AC-15, Santa Cruz Biotechnology). On the next day, the membranes were washed three times in TBST before incubation with the corresponding secondary antibodies for one hour at room temperature. The secondary antibodies were horseradish peroxidase-conjugated goat anti-rabbit, goat anti-mouse or donkey anti-sheep IgG antibodies (111–035-045, 115–035-062, 113–035-147, Jackson ImmunoResearch). After incubation, the membranes were washed three times in TBST and then visualized using enhanced chemiluminescence (ECL + , Amersham Biosciences) and ChemiDoc MP Imaging System (Bio-Rad).

### Tissue staining for confocal imaging

C2C12 myotubes were fixed in 4% paraformaldehyde/PBS, rinsed and permeabilized with 0.1% Triton-X/PBS. The fixed samples were incubated in 1% BSA/PBS blocking solution with 1:200 sarcomeric α-actinin monoclonal antibody (MA1-22863, Invitrogen) at room temperature for 2 h. 1:200 Alexa Fluor Plus 488 conjugated goat anti-mouse IgG secondary antibody (A32723, Invitrogen) was applied together with 1:100 Alexa Fluor 647 conjugated phalloidin (A22287, Invitrogen) for F-actin staining in blocking solution for 2 h at room temperature. 4′,6-diamidino-2-phenylindole, dihydrochloride (DAPI) (62247, Thermo Scientific) was used as nuclear stain. Stained samples were then washed with PBS and mounted with ProLong Gold Antifade (P10144, Molecular Probes). Images were acquired with Nikon Eclipse Ti2 microscope and NIS-Elements software.

### Mechanical testing of hydrogels

Gelatin hydrogels were prepared and cross-linked as described above in a 10 mL plastic syringe, which was subsequently stored at 5˚C overnight. To extract gelatin samples, the filled syringe was cut manually with a scalpel into cylinders of 5–9 mm height. The gelatin samples were carefully removed with a spatula and stored in a closed petri dish with PBS to avoid dehydration. Height and diameter of each sample were measured with a Digital Vernier Caliper Micrometer before performing a uniaxial compression test with an Instron 5967 using a 50 N load cell. The shear modulus was calculated using Neo-Hookean rubber theory, which predicts a linear relationship between stress and shear modulus, ${\sigma }_{1}=G\left({\lambda }_{1}-{\lambda }_{1}^{-2}\right)$, where (${\sigma }_{1})$ is stress, G is shear modulus and ${\lambda }_{1}$ is the extension ratio. A linear fit of the data for 1–10% compression was used for each sample to deduce the shear modulus. Young’s modulus (*E*) was calculated using the relation $E=2G*(\nu +1)$. A Poisson’s ratio of $\nu =0.5$ was applied, as we assumed incompressibility.

### Statistical analysis

The data were analyzed for normality using Saphiro–Wilk test. Western blot data were normally distributed and were analyzed using one-way or two-way ANOVA followed Tukey’s or by Sidak’s post hoc tests for multiple comparison, respectively. Alignment data were normally distributed and analyzed using one-way ANOVA followed by Bonferroni corrected pairwise t-test for multiple comparisons. Data which were not normally distributed were analyzed using the Kruskal–Wallis test followed by a Bonferroni corrected Wilcoxon test for multiple comparisons. Analyses were performed using either R or Graphpad Prism.

## Supplementary information

Supplementary figures

Supplementary movie 1

Supplementary movie 2

Supplementary movie 3

Supplementary movie 4
