# Supplementary material for: Large-scale spontaneous self-organization and maturation of skeletal muscle tissues on ultra-compliant gelatin hydrogel substrates
Source: Sci Rep. 2020 Aug 6;10:13305. doi: 10.1038/s41598-020-69936-6 (PMC7411013; doi:10.1038/s41598-020-69936-6)
Supplement: Supplementary file 1 — Supplementary figures [file 41598_2020_69936_MOESM1_ESM.docx]

***Supplementary Information***

***Large-scale spontaneous self-organization and maturation of skeletal muscle tissues on ultra-compliant gelatin hydrogel substrates***

Joen H. Jensen^1^, Selgin D. Cakal^1^, Jingwen Li^2^, Christian J. Pless^1^, Carmen Radeke^1^, Morten Leth Jepsen^1,3^, Thomas E. Jensen^2^, Martin Dufva^1, 3,^*, Johan U. Lind^1,^*

^1^ Department of Health Technology, Technical University of Denmark, 2800 Kgs. Lyngby, DK-Denmark

^2^ Section of Molecular Physiology, Department of Nutrition, Exercise and Sports, University of Copenhagen, 2100 København Ø, DK-Denmark

^3^ The Danish National Research Foundation and Villum Foundation's Center for Intelligent Drug Delivery and Sensing Using Microcontainers and Nanomechanics (IDUN), Technical University of Denmark, 2800 Kgs. Lyngby, DK-Denmark

**
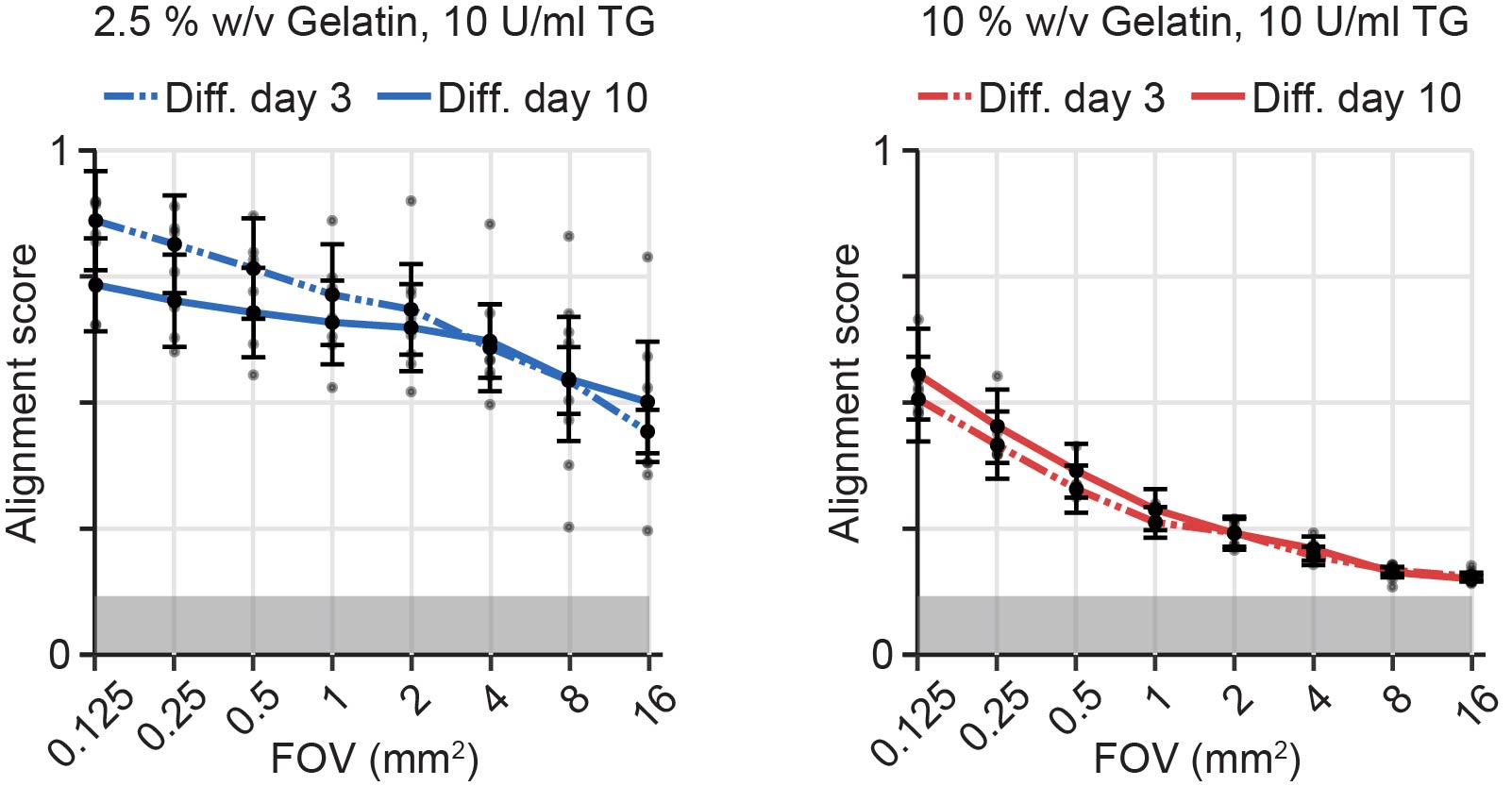
**

**Supplementary Figure 1 | Myotube alignment is stable during differentiation and maturation.** Local and global C2C12 myotube alignment given as alignment score (0-1) as a function of FOV, based on F-actin stains. Shaded area indicates values below theoretical point of random alignment (0.11). *Left:* C2C12 cultured on 2.5% w/v gelatin - 10 U/mL TG hydrogel substrates, evaluated at day 3 and 10 of differentiation. *Right:* C2C12 cultured on 10% w/v gelatin - 10 U/mL TG hydrogel substrates, evaluated at day 3 and 10 of differentiation. Markers indicate mean ± s.e.m. (n = 4), individual data points included as dots.

**
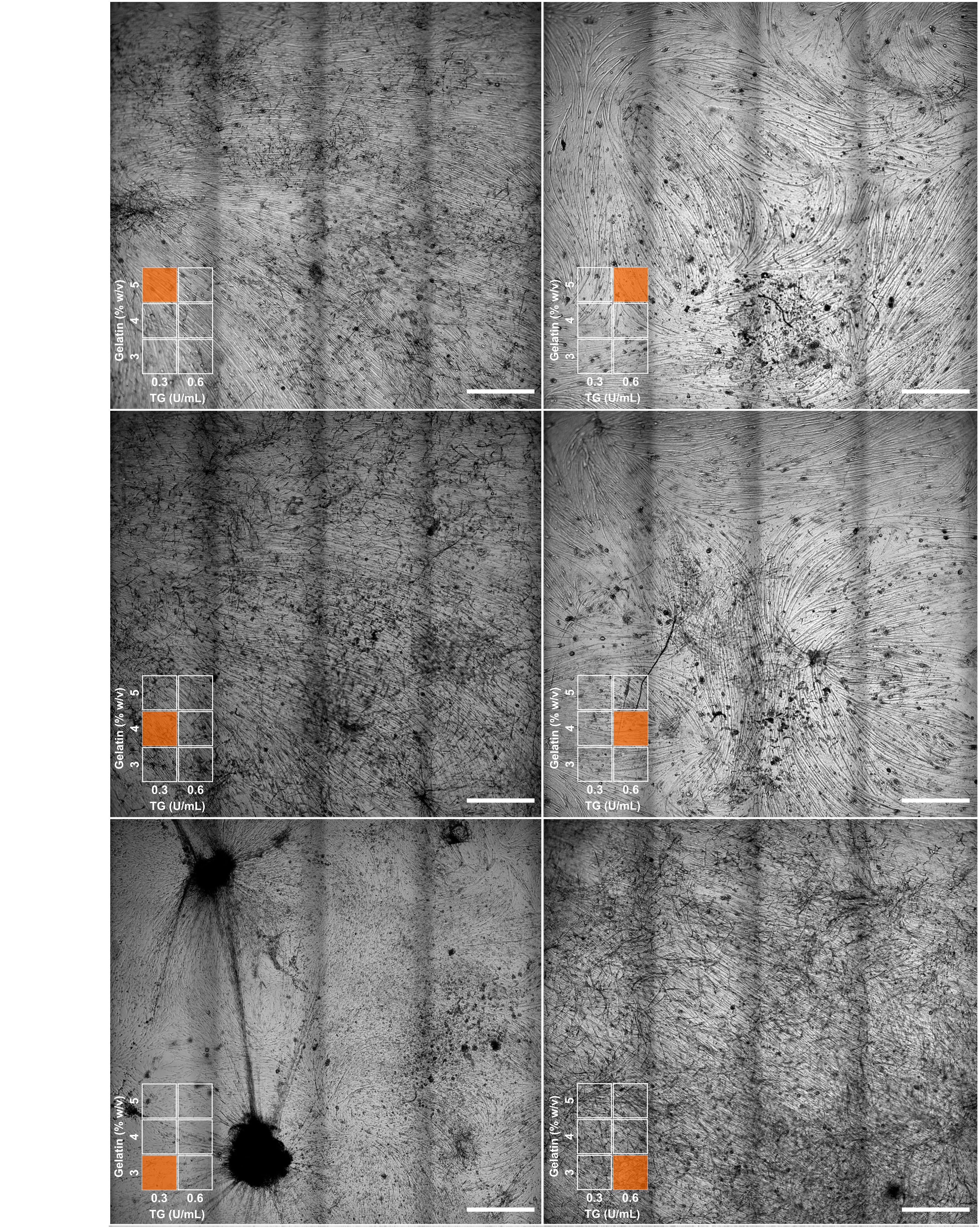
**

**Supplementary Figure 2 | Myotube cultures on additional low concentration gelatin hydrogel formulations**

Bright-field microscopy images of C2C12 myotubes cultured on 3, 4 and 5 % w/v gelatin hydrogel substrates cross-linked using 0.3 or 0.6 U/ml transglutaminase (TG). Images obtained at day 9 after initiation of differentiation. Scale-bars: 1 mm

**
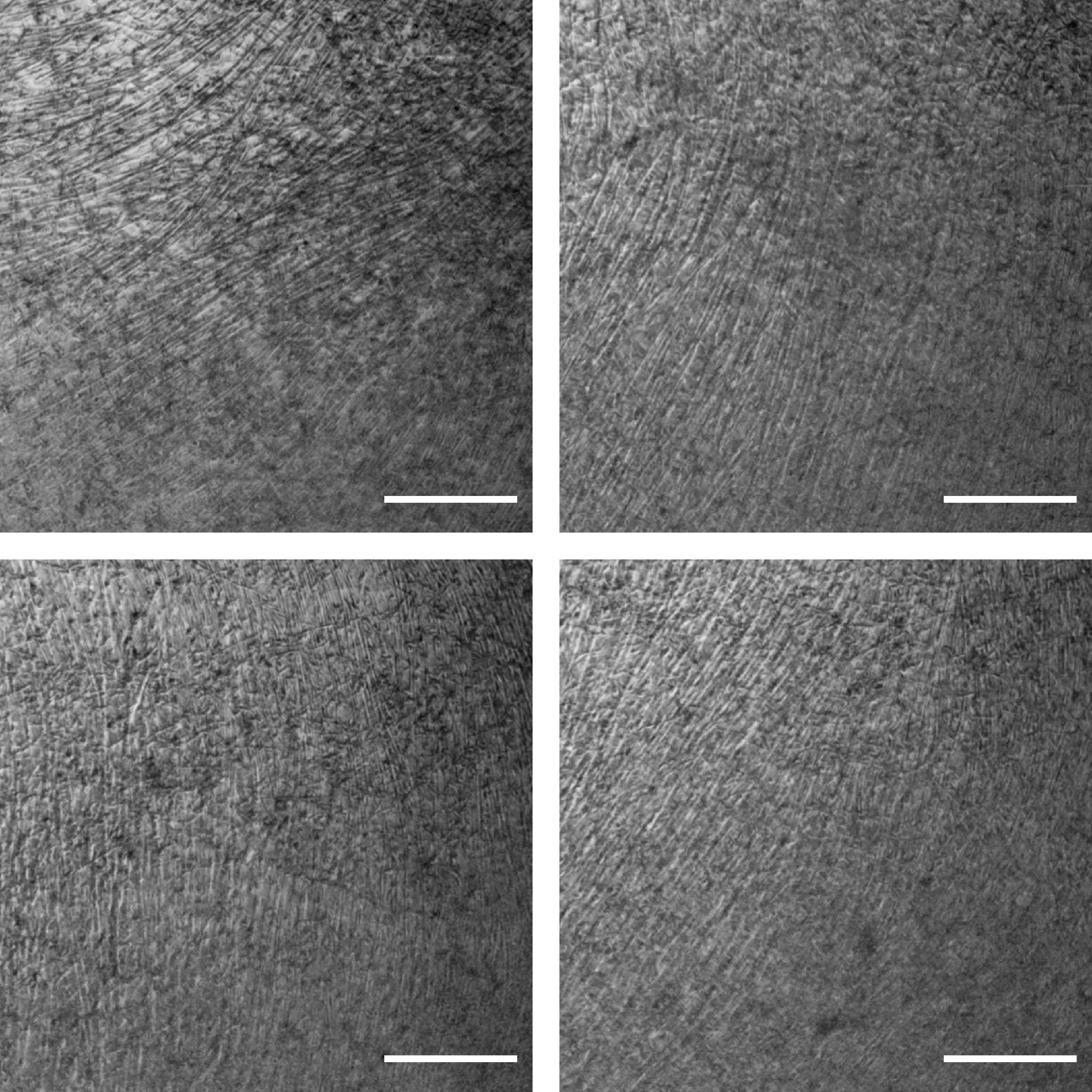
**

## Supplementary Figure 3 | Spontaneous alignment of separate C2C12 strain. Bright field microscopy images of a separate C2C12 strain (passage 9) cultured on 2.5 % w/v gelatin hydrogel substrates cross-linked using 10 U/ml transglutaminase (TG). Images obtained at day 17 of differentiation. Scale-bars 0.5mm.

**
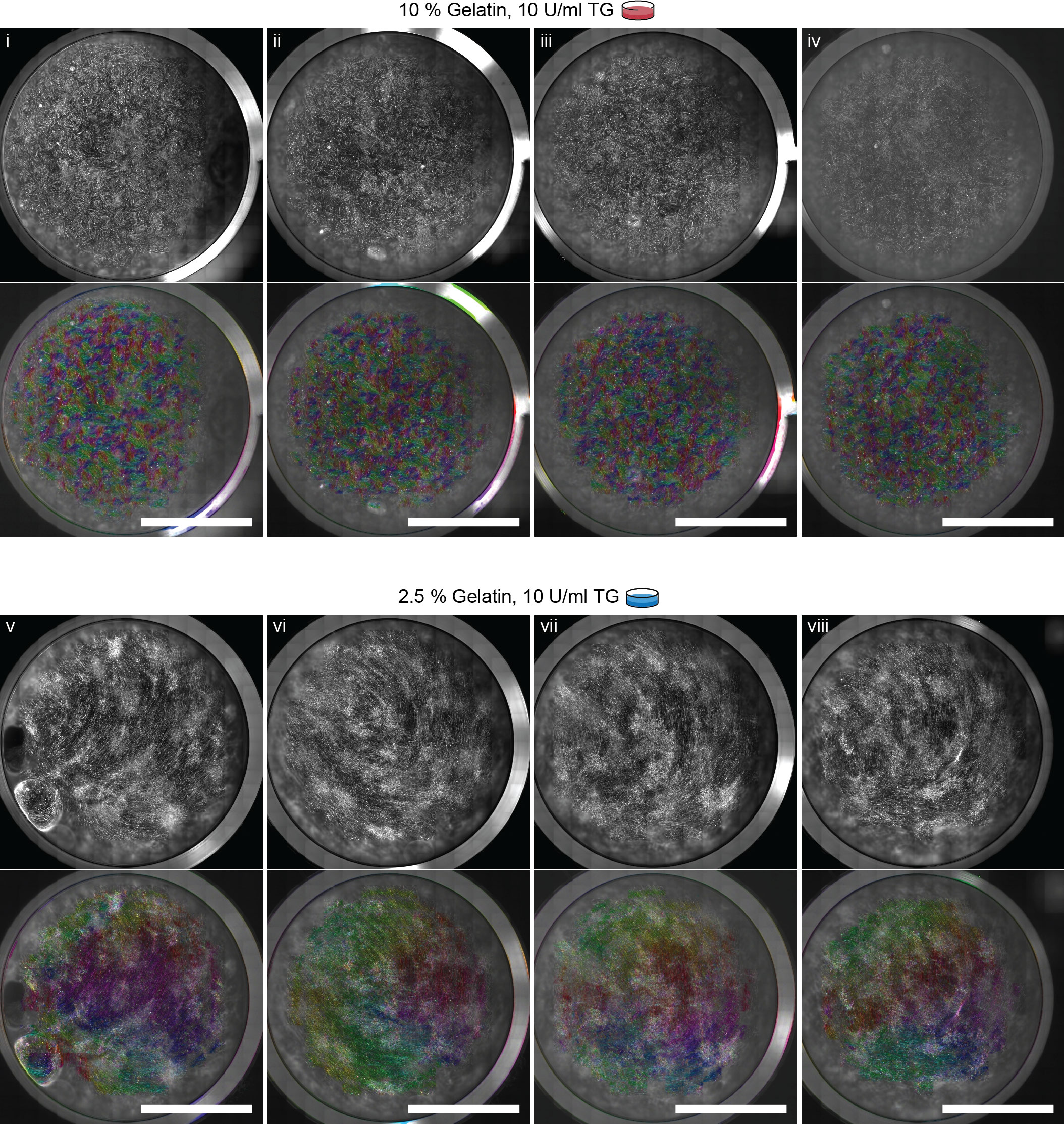
**

**Supplementary Figure 4 | Large-scale self-organization is consistent.** Composite images of F-actin in C2C12 myotubes, spanning full wells in a standard 12-well plate. Composite images were composed of 252 frames, and the orientationJ plug-in was used to false-color the composite image according to the orientation of the F-actin. *Top rows:* C2C12 culture on 10% w/v gelatin - 10 U/mL TG hydrogels. *Bottom rows:* C2C12 culture on 2.5% w/v gelatin-10 U/mL TG hydrogels. Images obtained at day 9 after initiation of differentiation. Scale-bars 10 mm

**
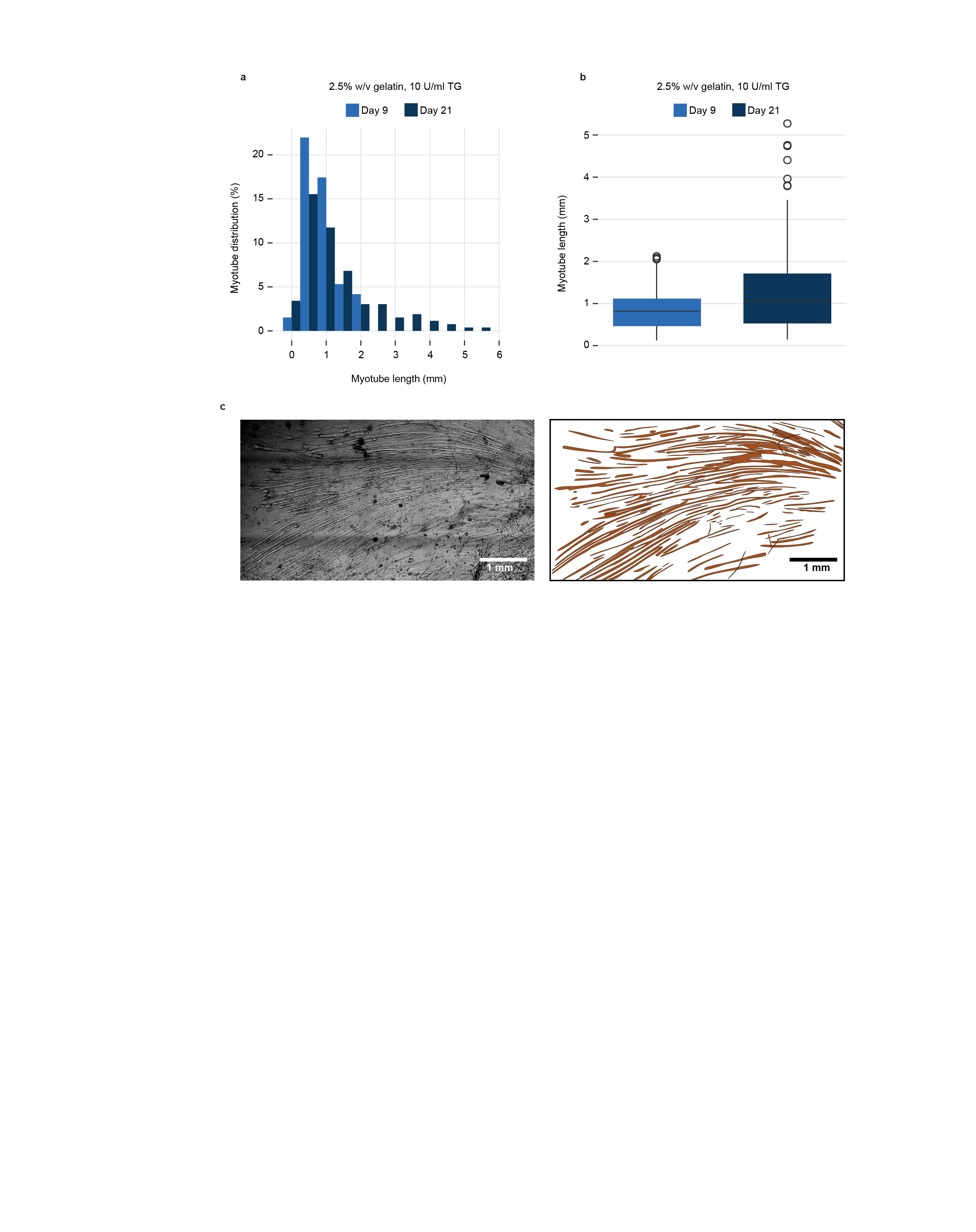
**

**Supplementary Figure 5 | Myotubes can reach physiological length scales.** Myotube length distribution for C2C12 culture grown on 2.5% w/v gelatin - 10 U/mL TG at 9 and 21 days of differentiation. **(a)** Histogram over myotube lengths shows that at day 21 of differentiation, myotubes of up to more than 5 mm have formed **(b)** Same data show as a box plot. Middle bar represents median length, while the upper and lower bound of the box respectively represents the upper and lower quartile. Lines indicate largest and lowest value within 1.5 times the interquartile range, while circles indicate outliers (outside 1.5 interquartile ranges of the median). The size of the field of view used to make these quantifications differed (3.4 mm x 2.7 mm FOV for day 9 and 15.8 mm x 11.9 mm for day 21), which will in turn results in a slight underestimation of day 9 myotube lengths. In spite of this systematic discrepancy, it is evident from the graphs that myotube sizes increases beyond 9 days of differentiation on ultra soft gelatin hydrogels. **(c)** Example images of 21 day samples and visualization of analysis.


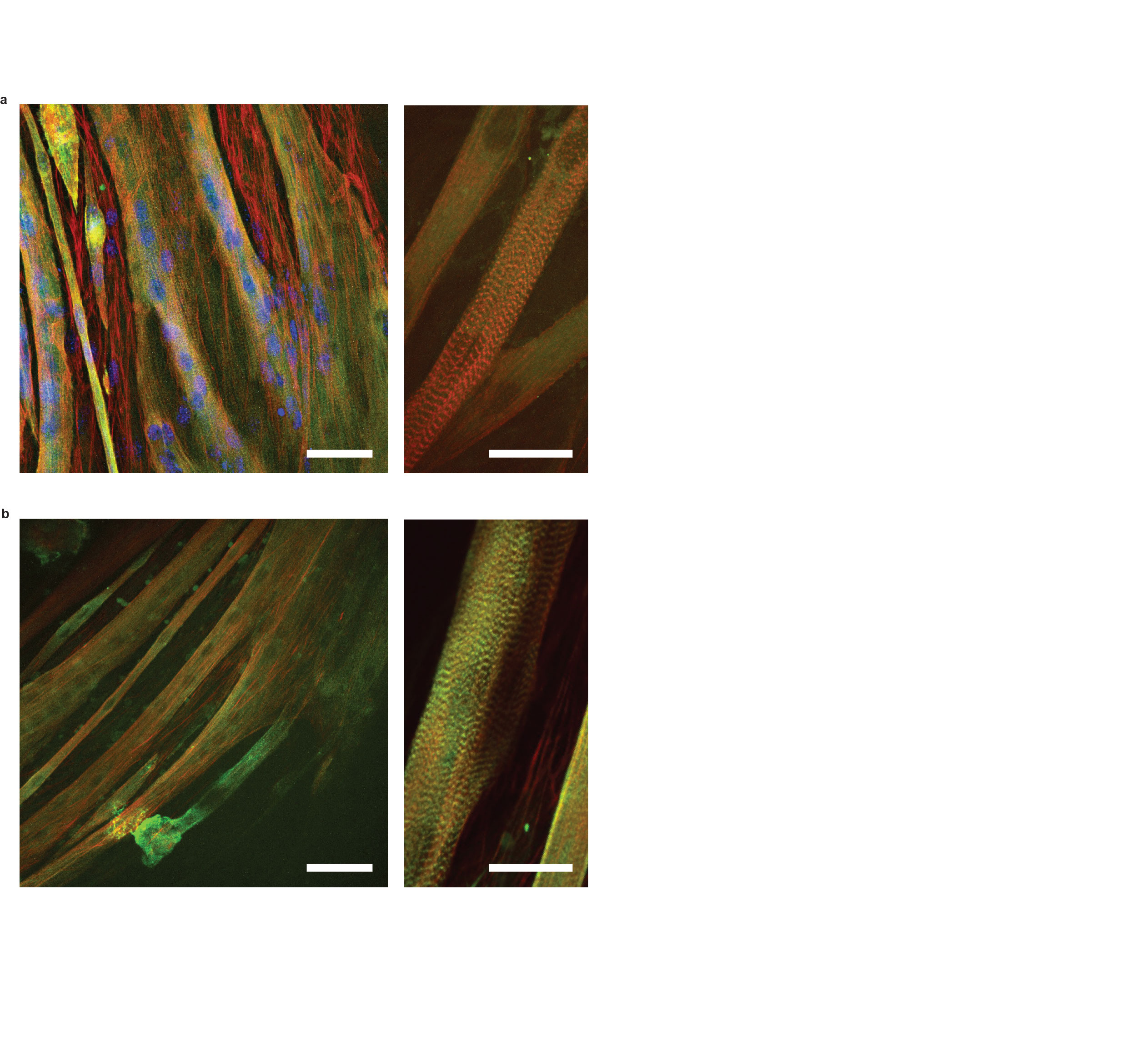


**Supplementary Figure 6 | Striated myotubes on gel substrates.** Confocal images of immunostained myotubes at differentiation day 21. *Blue:* DAPI, *Red:* Actin, *Green:* Alpha-actinin. Scale-bars *left:* 50 µm, *Right:* 25 µm. **a)** 5% w/v gelatin - 0.6 U/ml TG hydrogel substrate, **b)** 10% w/v gelatin - 10 U/ml TG hydrogel substrate.

**
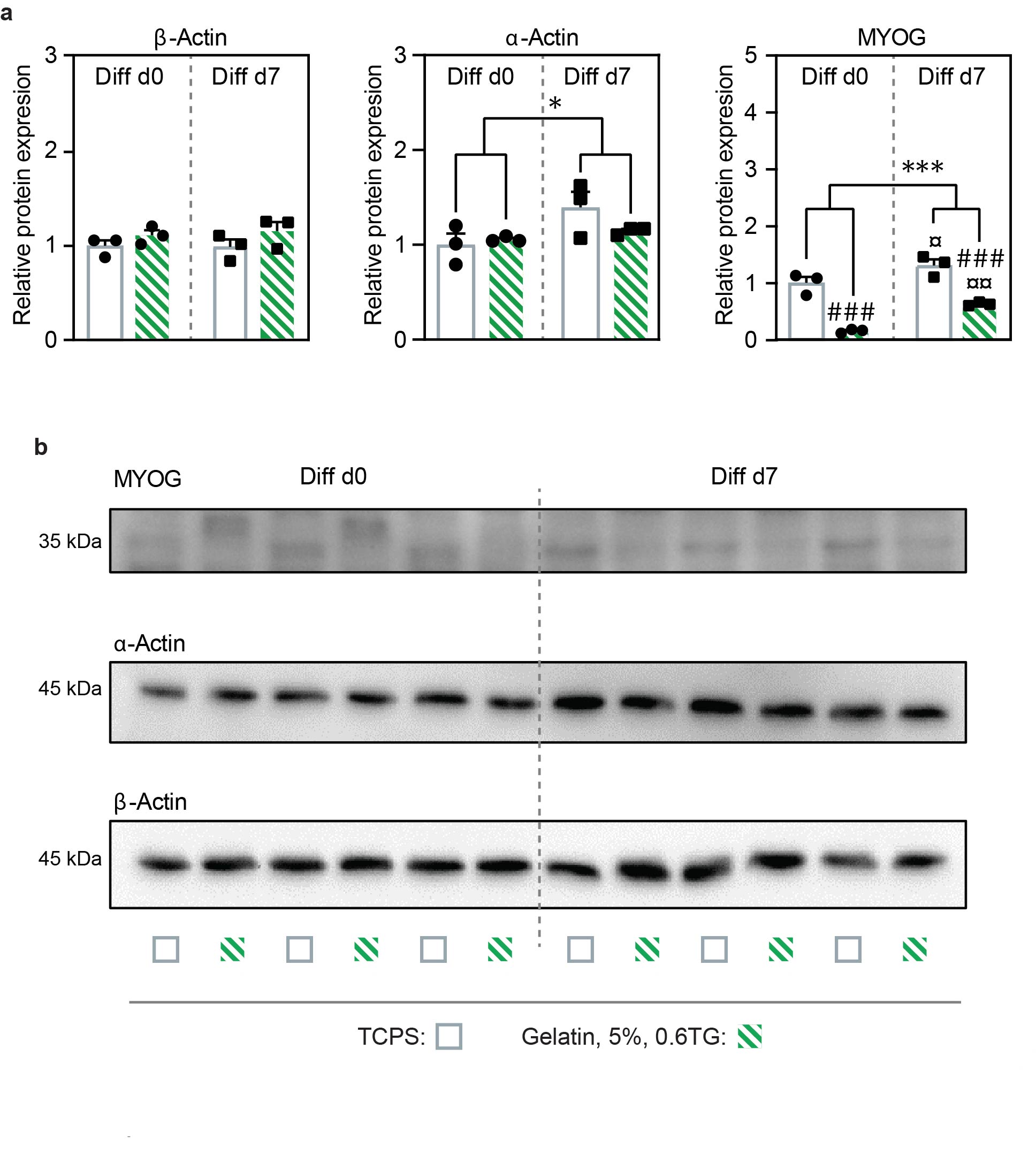
**

**Supplementary Figure 7 |** **Myogenin expression is diminished on gel substrates before and after initiation of differentiation.** Western-blot evaluation of C2C12 myoblast samples immediately before initiation of differentiation, and myotubes samples after 7 days of differentiation, for TCPS vs 5% w/v gelatin – 0.6 U/ml TG. For each protein, quantification of expression changes relative to protein content of day 0 differentiation TCPS is shown. Error bars indicate mean ± s.e.m. Western blot trace depicted at bottom. Full gel images shown in Supp. Figures 13-15. A Two-way ANOVA followed by Sidak’s post hoc test was performed. Statistical significance indicators: * p<0.05, ** p<0.01, *** p<0.001, significant difference between day 0 and day 7 differentiation; ¤ p<0.05, ¤¤ p<0.01, significant difference between day 0 and day 7 differentiation for given substrate; ### p<0.001, significant difference between substrates within day of differentiation.

**
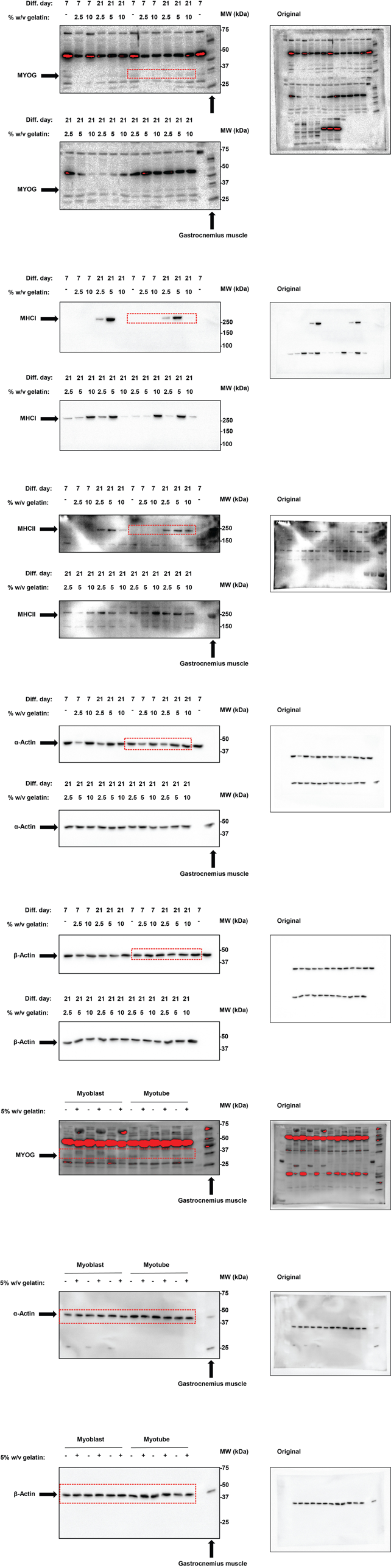
**

**Supplementary Figure 8 | Raw Western blot data for evaluation of MYOG presented in Figure 4.** Each band represent an independent replicate, which was used for quantification**.** Highlighted box indicated cropped example section displayed in Figure 4. Gastrocnemius muscle was used as positive control.

**
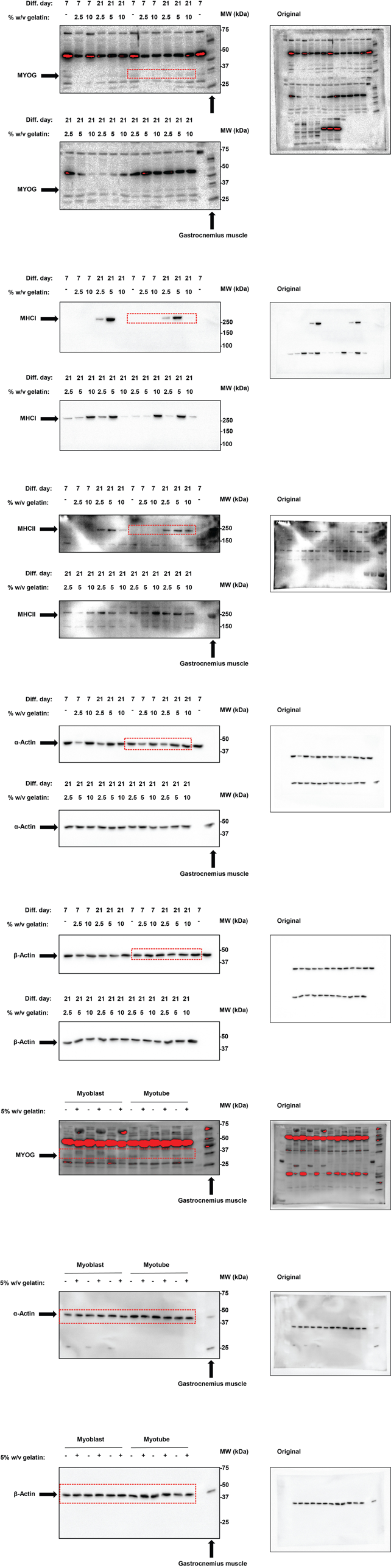
**

**Supplementary Figure 9 | Raw Western blot data for evaluation of MyHCI presented in Figure 4.** Each band represent an independent replicate, which was used for quantification**.** Highlighted box indicated cropped example section displayed in Figure 4. Gastrocnemius muscle was used as positive control.

**
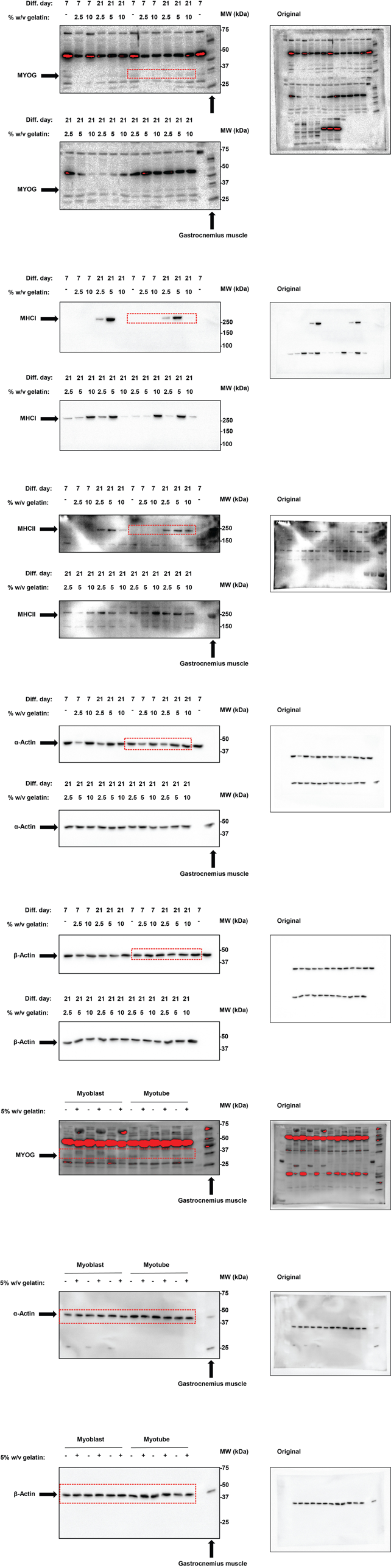
**

**Supplementary Figure 10 | Raw Western blot data for evaluation of MyHCII presented in Figure 4.** Each band represent an independent replicate, which was used for quantification**.** Highlighted box indicated cropped example section displayed in Figure 4. Gastrocnemius muscle was used as positive control.

**
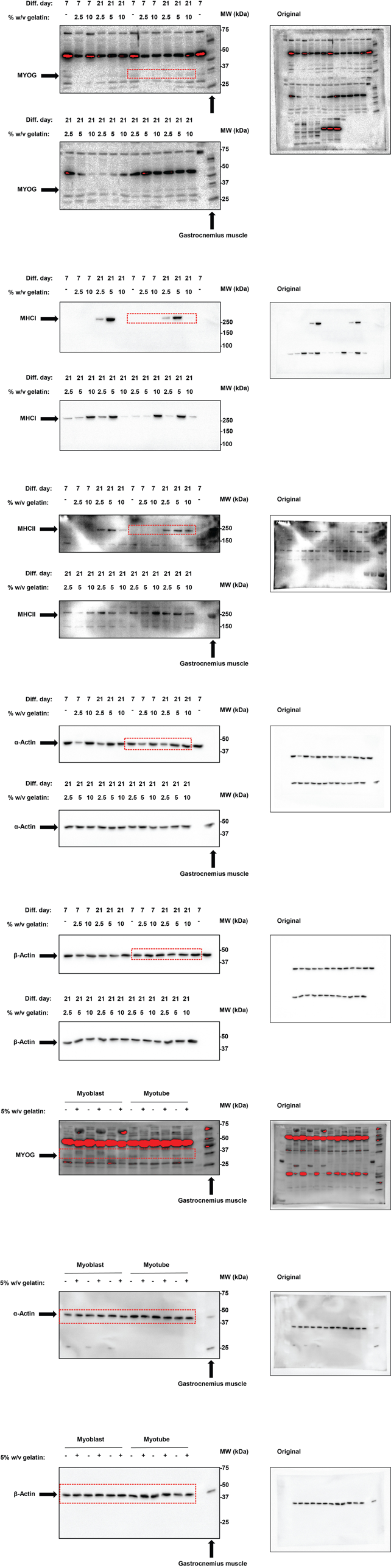
**

**Supplementary Figure 11 | Raw Western blot data for evaluation of α-actin presented in Figure 4.** Each band represent an independent replicate, which was used for quantification**.** Highlighted box indicated cropped example section displayed in Figure 4. Gastrocnemius muscle was used as positive control.

**
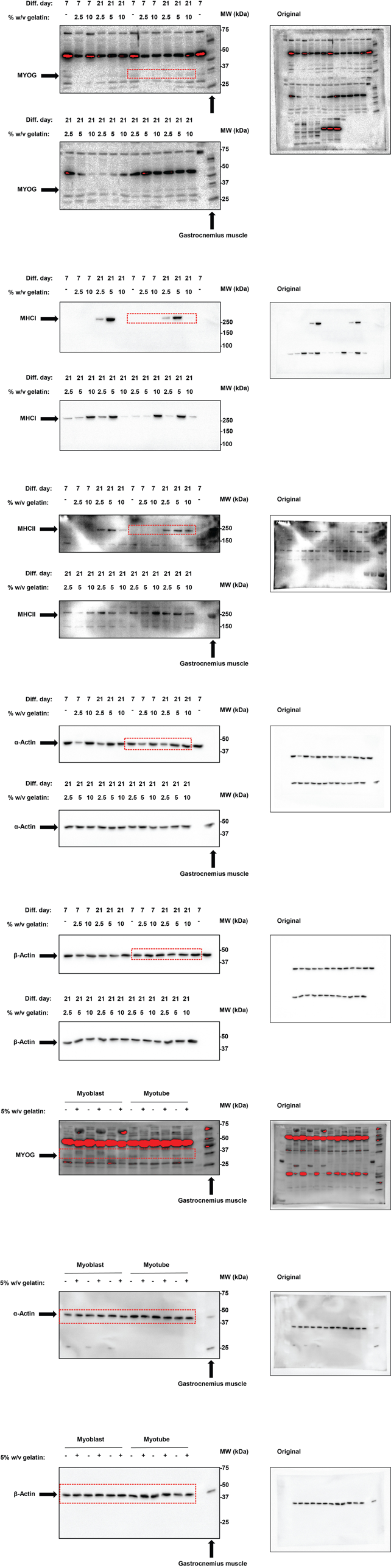
**

**Supplementary Figure 12 | Raw Western blot data for evaluation of β-actin presented in Figure 4.** Each band represent an independent replicate, which was used for quantification**.** Highlighted box indicated cropped example section displayed in Figure 4. Gastrocnemius muscle was used as positive control **
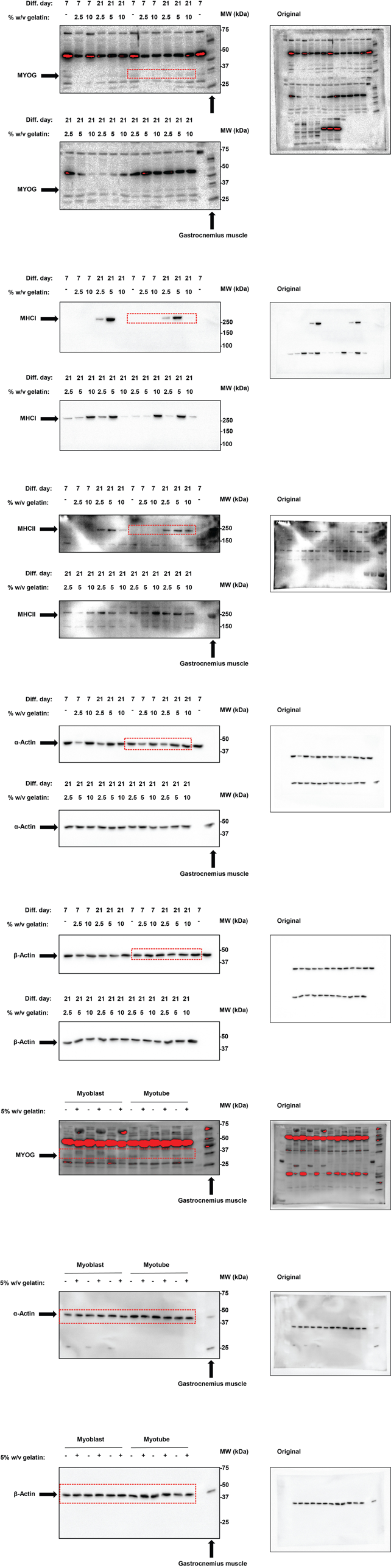
**

**Supplementary Figure 13 | Raw Western blot data for evaluation of MYOG presented in Supp. Figure 7.** Each band represent an independent replicate, which was used for quantification**.** Highlighted box indicated cropped example section displayed in Supp. Figure 7. Gastrocnemius muscle was used as positive control **
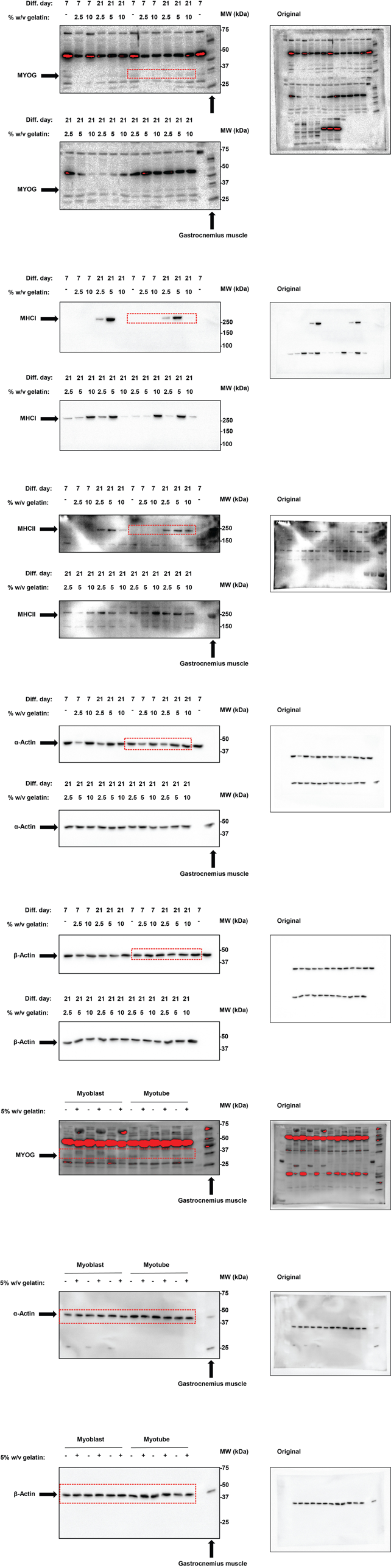
**

**Supplementary Figure 14 | Raw Western blot data for evaluation of α-actin presented in Supp. Figure 7.** Each band represent an independent replicate, which was used for quantification**.** Highlighted box indicated cropped example section displayed in Supp. Figure 7. Gastrocnemius muscle was used as positive control

**
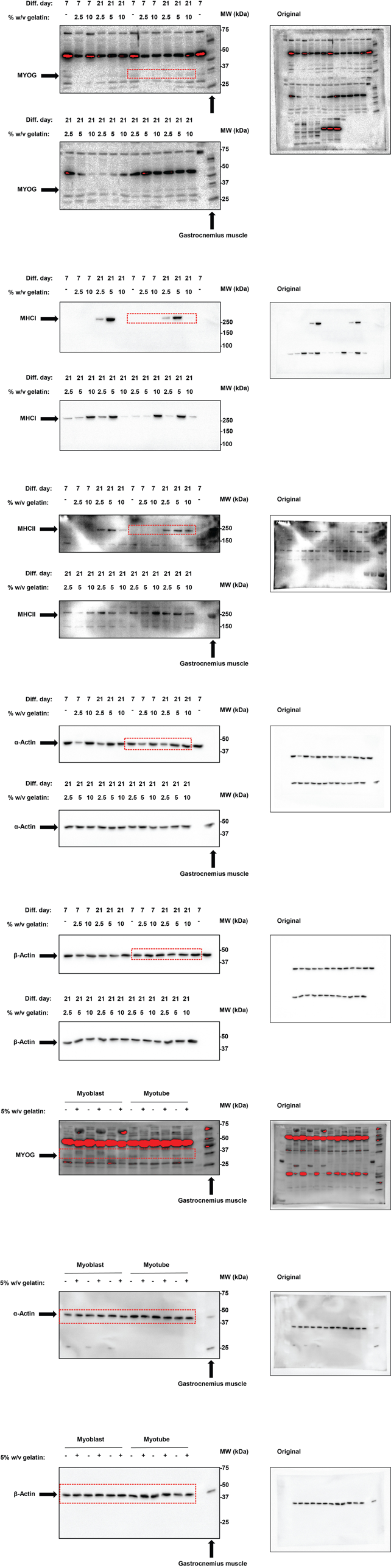
**

**Supplementary Figure 15 | Raw Western blot data for evaluation of β-actin presented in Supp. Figure 7.** Each band represent an independent replicate, which was used for quantification**.** Highlighted box indicated cropped example section displayed in Supp. Figure 7. Gastrocnemius muscle was used as positive control

**List of Supplementary Movies**

Movie 1: Spontaneous contraction of C2C12 myotubes developed on 2.5 % w/v gelatin - 10 U/ml TG hydrogel at day 12 of differentiation

Movie 2: Spontaneous contraction of C2C12 myotubes developed on 5 % w/v gelatin – 0.6 U/ml TG hydrogel at day 12 of differentiation

Movie 3: Spontaneous contraction of C2C12 myotubes developed on 10 % w/v gelatin - 10 U/ml TG hydrogel at day 12 of differentiation

Movie 4: Spontaneous tetanus contractions of C2C12 myotubes developed on 2.5 % w/v gelatin - 10 U/ml TG hydrogel at day 14 of differentiation
